# Application of SHAP values for inferring the optimal functional form of covariates in pharmacokinetic modeling

**DOI:** 10.1002/psp4.12828

**Published:** 2022-06-24

**Authors:** Alexander Janssen, Mark Hoogendoorn, Marjon H. Cnossen, Ron A. A. Mathôt, for the OPTI‐CLOT Study Group and SYMPHONY Consortium, M. H. Cnossen, S. H. Reitsma, F. W. G. Leebeek, R. A. A. Mathôt, K. Fijnvandraat, M. Coppens, K. Meijer, S. E. M. Schols, H. C. J. Eikenboom, R. E. G. Schutgens, E. A. M. Beckers, P. Ypma, M. J. H. A. Kruip, S. Polinder, R. Y. J. Tamminga, P. Brons, K. Fischer, K. P. M. van Galen, F. C. J. I. Heubel‐Moenen, L. Nieuwenhuizen, M. H. E. Driessens, I. van Vliet, J. Lock, H. C. A. M. Hazendonk, I. van Moort, J. M. Heijdra, M. H. J. Goedhart, W. Al Arashi, T. Preijers, N. C. B. de Jager, L. H. Bukkems, M. E. Cloesmeijer, A. Janssen, P. W. Collins, R. Liesner, P. Chowdary, C. M. Millar, D. Hart, D. Keeling

**Affiliations:** ^1^ Department of Clinical Pharmacology, Hospital Pharmacy Amsterdam University Medical Center Amsterdam The Netherlands; ^2^ Quantitative Data Analytics Group, Department of Computer Science, Vrije Universiteit Amsterdam Amsterdam The Netherlands; ^3^ Department of Pediatric Hematology, Erasmus MC Sophia Children’s Hospital Erasmus University Medical Center Rotterdam The Netherlands

## Abstract

In population pharmacokinetic (PK) models, interindividual variability is explained by implementation of covariates in the model. The widely used forward stepwise selection method is sensitive to bias, which may lead to an incorrect inclusion of covariates. Alternatives, such as the full fixed effects model, reduce this bias but are dependent on the chosen implementation of each covariate. As the correct functional forms are unknown, this may still lead to an inaccurate selection of covariates. Machine learning (ML) techniques can potentially be used to learn the optimal functional forms for implementing covariates directly from data. A recent study suggested that using ML resulted in an improved selection of influential covariates. However, how do we select the appropriate functional form for including these covariates? In this work, we use SHapley Additive exPlanations (SHAP) to infer the relationship between covariates and PK parameters from ML models. As a case‐study, we use data from 119 patients with hemophilia A receiving clotting factor VIII concentrate peri‐operatively. We fit both a random forest and a XGBoost model to predict empirical Bayes estimated clearance and central volume from a base nonlinear mixed effects model. Next, we show that SHAP reveals covariate relationships which match previous findings. In addition, we can reveal subtle effects arising from combinations of covariates difficult to obtain using other methods of covariate analysis. We conclude that the proposed method can be used to extend ML‐based covariate selection, and holds potential as a complete full model alternative to classical covariate analyses.

**Study Highlights**

**WHAT IS THE CURRENT KNOWLEDGE ON THE TOPIC?**

Covariate selection in pharmacokinetic (PK) modeling is a complex process and is sensitive to bias. Machine‐learning (ML) techniques might help to simplify and potentially improve this process, but are difficult to interpret as is.

**WHAT QUESTION DID THIS STUDY ADDRESS?**

Can we use ML models to infer the optimal functional form to represent the relationship between covariates and PK parameters using SHapley Additive exPlanations?

**WHAT DOES THIS STUDY ADD TO OUR KNOWLEDGE?**

This study presents an extension to covariate selection procedures using ML methods. The resulting framework allows for the detection of intricate effects of covariates, which far exceed the capabilities of classical (linear) covariate analyses. In addition, it is more flexible with respect to covariate importance scores generally used in ML‐based covariate selection.

**HOW MIGHT THIS CHANGE DRUG DISCOVERY, DEVELOPMENT, AND/OR THERAPEUTICS?**

By learning the optimal functional form of covariates based on data the complexity of covariate selection and implementation is reduced. This accelerates PK model development and might help improve the accuracy of PK parameter estimates.

## INTRODUCTION

In population pharmacokinetic (PK) modeling, identification of the relationship between PK parameters and covariates is important for the explanation of interindividual variation (IIV). The classic stepwise method is among the most popular methods but is not without flaws. In stepwise methods, covariate selection is determined by a significant change in the objective function value following inclusion or exclusion of each covariate. Due to the ordered nature of this process, the method may suffer from bias and multiplicity issues.^1^, ^2^, ^3^

The full fixed effects model (FFEM), which is based on a full model fit, was introduced to reduce selection bias.^4^ In this method, all covariates of interest are tested simultaneously and included if they result in a clinically relevant change of the typical PK parameters. Although an improvement over the stepwise method, the FFEM is not able to solve all prior issues. In both methods, an assumption must be made about the functional form describing the relationship between the covariate and the PK parameters. This encourages data dredging because various functional forms can be tested until one satisfies the criteria for inclusion. Furthermore, true covariates may be excluded when suboptimal functional forms are used. In summary, we identify a need for a covariate selection method which performs both a full model fit, while simultaneously estimating the optimal functional form of each covariate.

A recent study describes the use of machine learning (ML) for performing covariate selection for PK models.^5^ Here, the authors discuss how combining ML algorithms with covariate importance scores can be used to obtain a similar or better selection of covariates compared to stepwise methods. Other studies further discuss using such an approach on real‐life data to obtain a set of predictive covariates.^6^, ^7^ ML algorithms might be suitable for this task as they can learn covariate relationships directly from data. These methods might thus reduce the issue of selecting suboptimal functional forms when testing covariates for inclusion. Many ML software packages provide measures of covariate importance. For tree‐based methods (e.g., random forests^8^ or gradient boosting trees^9^), examples include counting the number of uses of each covariate, or more sophisticated measures, such as Gini or permutation importance. Although often found to be relatively accurate, there are situations where these measures may be biased.^10^ In addition, they only provide a single score of importance without information about the relationship between each covariate and model output. After obtaining a set of important covariates, how do we now select the functional form to implement these covariates without again resorting to stepwise methods?

SHapley Additive exPlanations (SHAP) is a promising model explanation technique due to its strong theoretical base.^11^ In addition to a more robust benchmark performance compared to other approaches,^12^ SHAP allows for identification of the influence of specific covariates and their effect on each individual prediction. The use of SHAP might improve upon importance scores by also allowing for the analysis of the relationship between covariates and model output. Its use for covariate selection has, however, not yet been explored.

In this study, we will focus on tree‐based ML algorithms, as there exists an exact method for the computation of SHAP values for these types of models.^12^ Specifically, we will use the random forest and XGBoost algorithms.^13^ Both methods create an ensemble model of decision trees. A decision tree is an algorithm that groups observations into bins (appropriately called leaves), which share a similar value for the response variable. Each tree is composed of multiple layers, where the observation is split into two leaves based on the value of one of the covariates. In a random forest, the model prediction is averaged over multiple independently fit trees. Each tree is fit using a subset of the data adding stochasticity to the learning process aiming to reduce overfitting. In gradient boosting trees (e.g., XGBoost), the trees are built sequentially, so that additional decision trees are added if they improve the prediction of the previous model ensemble. Each tree is thus fit to improve the mistakes of the previous tree. The objective function also contains a regularization term which penalizes the addition of complex models. In contrast to the classic random forest implementation, XGBoost supports missing values.^13^

Our goal is to evaluate the value of combining ML and SHAP for enriching ML‐based covariate analysis in the context of PK models. To this end, we will fit a random forest and XGBoost model to predict empirical Bayes estimates of PK parameters and perform a SHAP analysis on the most accurate model. As a case study, we use a retrospective dataset of patients with hemophilia A receiving clotting factor VIII (FVIII) while undergoing surgery.^14^ We explore the output of the SHAP analysis and present how it can be used for understanding the relationship between covariates and PK parameters.

## METHODS

### Dataset

We used retrospective data of 119 individuals with hemophilia A undergoing surgery in five different hemophilia treatment centers in the Netherlands.^14^ Patients received clotting factor FVIII concentrate (via bolus or continuous doses) to reach target FVIII levels as set by the Dutch National Hemophilia Consensus. This guideline recommends the following FVIII peak levels during the peri‐operative window: 0.80–1.00 IUml^−1^ at 0–24 h, 0.50–0.80 IUml^−1^ at 24–120 h, and 0.30–0.50 IUml^−1^ beyond 120 h post‐surgery. A total of 3350 FVIII levels were measured during 197 surgical procedures. All FVIII levels were measured using the one‐stage clotting assay. Timing and dosage of measurements was determined at the discretion of the treating physician. For most patients, this resulted in more frequent measurements early in the peri‐operative window, and occasional measurements post‐surgery to validate if the patient still met target levels. The following 13 covariates were chosen for analysis: treatment center (1–5), pre‐assessed surgical risk (low vs. high^15^), use of β‐domain deleted recombinant FVIII (BDD‐FVIII, moroctocog alfa/Refacto AF), hemophilia severity (moderate vs. severe), FVIII baseline levels, blood group (O vs. non‐O), blood loss during surgery, occurrence of a bleeding complication, body weight, body mass index (BMI), age in years, and von Willebrand factor antigen (VWF:Ag) and activity (VWF:act) levels. Five covariates contained missing values. Missing values were either imputed by mean (for continuous variables) or addition of a separate category (for categorical variables).

### Prediction of PK parameters using machine learning

Empirical Bayes estimates of the PK parameters were obtained by fitting a base two‐compartment model to the data using NONMEM (ICON Development Solutions, Ellicott City, MD^16^). Random effects were only estimated for the clearance and central volume parameters in order to improve model stability. A combined additive and proportional error model was used. We fixed the residual error estimates to $\sigma_{1}=0.08$ (additive error) and $\sigma_{2}=0.17$ (proportional error) to improve model stability and shrinkage while matching earlier findings.^17^, ^18^ Random forest (Python scikit‐learn package, version 0.23.2) and XGBoost (Python xgboost package, version 1.4.2) models were fit to predict the empirical Bayes estimated clearance and central volume distribution parameters independently. We fit the XGBoost models to both the original (containing missing values) and imputed data set. We performed a 10‐fold cross‐validation for the estimation of test set error and for SHAP value calculation. Default model hyperparameters were used (see Material S1 for details). Model accuracy was represented as the average mean absolute error (MAE) ± one SD of PK parameter predictions on the 10 test sets. We also calculated the root mean squared error (RMSE) of predicted FVIII levels by solving a two‐compartment model using the test set predicted PK parameters. The empirical Bayes estimated inter‐compartmental clearance and peripheral volume parameters were directly used for all patients. FVIII level predictions were performed in the Julia programming language (Julia Computing, Inc., version 1.6.0) using the DifferentialEquations package (version 6.17.1).^19^ The RMSE was presented as the mean and SD of the RMSE calculated for each individual patient.

### SHAP analysis

A SHAP analysis (Python shap package, version 0.36.0) was performed to explain model output. This method decomposes a model $f\left(x\right)$ into a simpler additive model:

$$f\left(x\right)=\phi_{0}+\sum_{i=1}^{M}\phi_{x_{i}}$$

Here, the SHAP value $\phi_{x_{i}}$ of covariate i∈M represents its direct effect on the model prediction, whereas $\phi_{0}$ represents the typical prediction. By cumulating the SHAP values for each individual, we can visualize their relationships with each of the covariates. For each of the 10 cross‐validations, we calculated SHAP values on the corresponding test set. The SHAP values were pooled and a smoothened representation of the effect was obtained by means of locally estimated scatterplot smoothing (LOESS; Python statsmodels package, version 0.12.2). SHAP values for missing continuous covariates were removed from visualizations.

### Model code

All model code, including implementation instructions, will be made available at https://github.com/Janssena/pkSHAP at the time of publication.

## RESULTS

### Patient characteristics and model accuracy

An overview of the patient characteristics, missing data, and the base model parameter estimates is shown in Table 1. RMSE of FVIII level predictions by the base nonlinear mixed effects (NLME) model was 0.23 IUml^−1^ ± 0.27 (SD). Accuracy of the ML models is depicted in Table 2. The MAE of PK parameter predictions by both ML algorithms fit to the imputed data set was similar. The XGBoost model fit to the original dataset resulted in higher MAE of both clearance (43.8 vs. 40.4 ml/h), as well as central volume predictions (893 vs. 807 ml) compared to the random forest model. In addition, the RMSE of the resulting FVIII level predictions was higher for the XGBoost model (0.36 vs. 0.32 IUml^−1^). The MAE of PK parameter predictions was indicative of the presence of residual IIV unexplained by the current set of covariates.

**TABLE 1:** Patient characteristics

	No. of procedures (%) or median [minimum‐maximum]	No. of missing data (%)
Covariates		
Weight, kg	75.0 [5–111]	0 (0)
Age, years	39.8 [0.24–77.7]	0 (0)
BMI	24.1 [13.6–32.8]	21 (10.7)
Treatment center		0 (0)
One	40 (20.3)	
Two	45 (22.8)	
Three	76 (38.6)	
Four	16 (8.1)	
Five	20 (10.2)	
Blood group		26 (13.2)
Non‐O	82 (41.6)	
O	80 (40.6)	
FVIII concentrate		3 (1.5)
BDD‐rFVIII	28 (14.2)	
Non BDD‐rFVIII	166 (84.3)	
High pre‐assessed surgical risk	97 (49.2)	0 (0)
Has severe hemophilia	147 (74.6)	0 (0)
Blood loss, ml	0 [0–6700]	0 (0)
Had bleeding complication	30 (15.2)	0 (0)
FVIII baseline level, IUml^−1^	0.0 [0.0–0.05]	0 (0)
VWF:Ag, %	120 [25–250]	79 (40.1)
VWF:Act, %	130 [24–270]	99 (50.3)
NLME model parameters		
CL, ml/h	163 [29.5–387]	
V1, ml	3030 [260–9710]	
Q, ml/h	56.9	
V2, ml	1270	
$\;\omega_{\mathrm{CL}}$ (%CV)	65.2	
$\omega_{V1}$ (%CV)	83.5	

Abbreviations: %CV, percent coefficient of variation; BDD‐rFVIII, β‐domain deleted recombinant clotting factor FVIII; BMI, body mass index; CL, clearance; NLME, nonlinear mixed effects; Q, intercompartmental clearance; V1, central volume; V2, peripheral volume; VWF:Act, von Willebrand factor activity; VWF:Ag, von Willebrand factor antigen.

**TABLE 2:** Accuracy of PK parameter and concentration predictions

	Random forest	XGBoost	XGBoost impute
MAE of CL predictions, ml/h	40.4 ± 10.5 SD (R^2^ = 0.56)	43.8 ± 10.8 SD (R^2^ = 0.48)	42.4 ± 11.0 SD (R^2^ = 0.50)
MAE of V1 predictions, ml	807 ± 320 SD (R^2^ = 0.49)	893 ± 356 SD (R^2^ = 0.37)	817 ± 308 SD (R^2^ = 0.47)
RMSE of concentration predictions, IUml^−1^	0.32 ± 0.20 SD	0.36 ± 0.26 SD	0.33 ± 0.22 SD

Abbreviations: CL, clearance; MAE, mean absolute error; PK, pharmacokinetic; RMSE, root mean squared error; SD, standard deviation; V1, central volume.

### SHAP analysis

**FIGURE 1:**
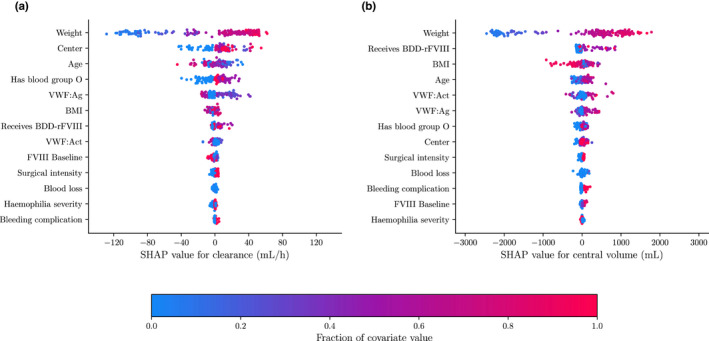
Overview of SHAP values for random forest model. SHAP values of the clearance (a) and central volume (b) are shown as calculated for the random forest model. The covariate value is indicated by color. The horizontal span of the SHAP values indicate the change in the parameters value. The larger the span, the larger the changes in PK parameter and thus the more important the covariate. Covariates are ranked from most (top) to least (bottom) influential by means of their mean absolute SHAP value. BDD‐rFVIII, β‐domain deleted recombinant clotting factor FVIII; BMI, body mass index; PK, pharmacokinetic; SHAP, SHapley Additive exPlanations; VWF, von Willebrand factor.

We present an overview of the SHAP values for the random forest models in Figure 1. This visualization can, for example, be used for the identification of influential covariates, as indicated by the horizontal span of SHAP values. Alternatively, we can use feature importance scores or the mean absolute SHAP value to rank the covariates based on influence. We have provided a comparison of these two scores in Figure S1. Both scores seem to lead to relatively similar results.

**FIGURE 2:**
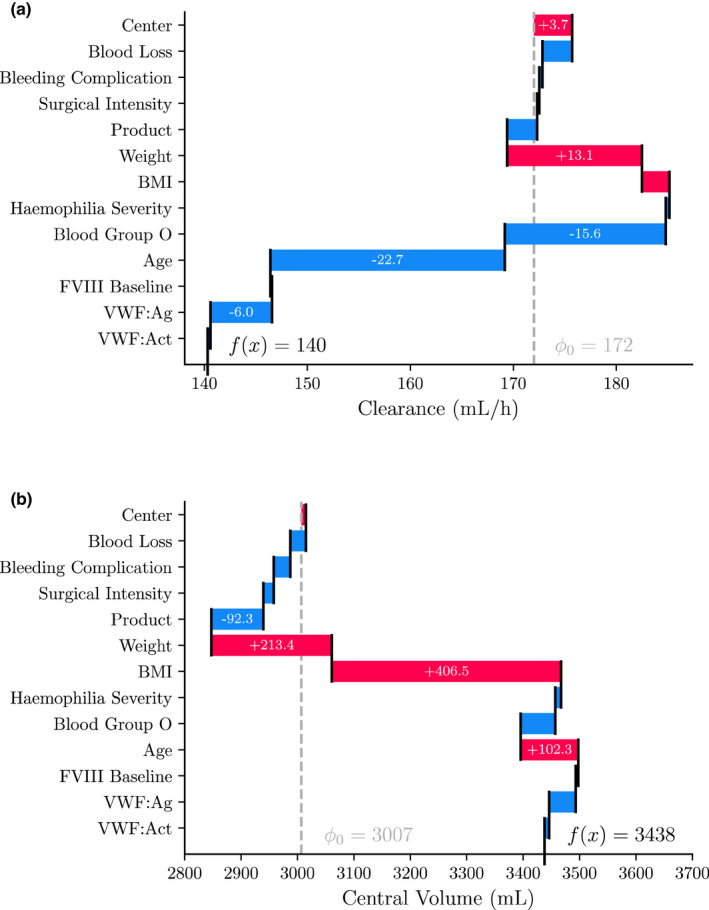
SHAP values for a typical patient. SHAP values are shown for the clearance (a) and central volume (b) predictions by the random forest. Data is shown for a 70 kg, 63 year old individual with blood group non‐O. SHAP value for each covariate is shown in the corresponding bar. Vertical dashed line indicates expected SHAP value. The SHAP values sum up to the final model prediction. BDD‐FVIII, β‐domain deleted clotting factor FVIII; BMI, body mass index; SHAP, SHapley Additive exPlanations; VWF, von Willebrand factor.

For both PK parameters, patient weight was the most influential covariate. For clearance (Figure 1a), treatment center, blood group, age, and VWF:Ag appeared to be relatively influential. For central volume (Figure 1b), BMI and use of BDD‐rFVIII concentrate seem to be the most important covariates aside from patient weight. The remaining covariates seem to be less influential for explaining the prediction. We can also take a look at the SHAP values for a single individual (Figure 2). Here, we can see the exact change in clearance and central volume resulting from the inclusion of each covariate.

**FIGURE 3:**
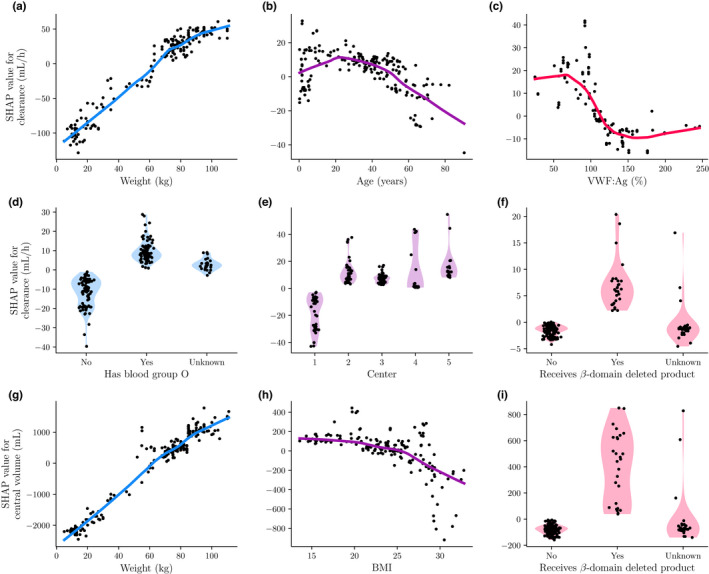
Relationship between covariates and PK parameters based on SHAP values. Here we visualize the relationship between PK parameter and covariate by plotting SHAP value against covariate value. Points represents the SHAP values, while lines indicate the LOESS fitted smooth representation of the relationship. For the categorical covariates the SHAP value density is also shown by means of a violin plot. We have shown the results for the most important covariates for clearance (a–f) and central volume (g–i). BMI, body mass index; LOESS, locally estimated scatterplot smoothing; PK, pharmacokinetic; SHAP, SHapley Additive exPlanations; VWF, von Willebrand factor.

Our main motivation for performing the SHAP analysis was the ability to visualize the relationship between the calculated SHAP values and each covariate of interest. In Figure 3, we present the resulting relationships for six covariates from the clearance model and three covariates from the central volume model. We observed a positive relationship between body weight and clearance, which flattened for weights above 65 kg (Figure 3a). For age, we saw a negative relationship with clearance, similar to earlier findings.^16^ We noticed that individuals with VWF:Ag levels below 100% had higher clearance than those with higher levels (Figure 3c). In addition, we observed that patients with blood group O displayed an increased clearance compared to non‐O individuals (Figure 3d). Both these findings were in line with physiological concepts of hemostasis. Next, we saw that the model predicts a decrease in clearance for individuals in center one, possibly as result of a confounder (Figure 3e). Finally, individuals who received a BDD‐rFVIII concentrate displayed slightly increased clearance compared to those who did not (Figure 3f).

For central volume, we also noted a positive relationship with body weight, which flattened slightly with increasing body weight (Figure 3g). We saw a sharp decrease in the SHAP values for central volume for individuals with a BMI 25 (ie, those classified as overweight; Figure 3h). Finally, we saw an increase in the SHAP values for individuals who received BDD‐rFVIII concentrate (Figure 3i).

**FIGURE 4:**
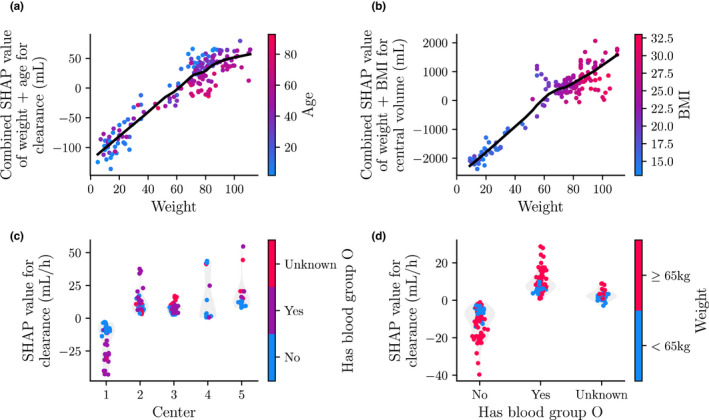
Interaction between SHAP values of the covariates. SHAP values of interactions between covariates are shown for the clearance (a, c, and d) and central volume (b) models. Points represents the SHAP values, while lines indicate the LOESS fitted smooth representation of the relationship. The value of the interacting covariate is indicated by color. For the categorical covariates the SHAP value density is also shown by means of a violin plot. BMI, body mass index; LOESS, locally estimated scatterplot smoothing; SHAP, SHapley Additive exPlanations.

We could further push the analysis by examining the combined effects of multiple covariates (Figure 4). Because body weight, BMI, and age were correlated, the true effect of either covariate might have been obscured by the others. We combined their respective SHAP values to determine if there was a unique effect of including the separate covariates. After this intervention, there were only small differences between the SHAP values of weight alone versus those of weight and BMI combined for clearance. The same was true for the combined SHAP values of weight and age for central volume. However, combining the SHAP values of weight and age for clearance showed that part of its variance could be well explained by age for individuals with a body weight above 65 kg (Figure 4a). Combining the SHAP values of weight and BMI for central volume resulted in a more pronounced flattening of SHAP values for individuals with a body weight above 65 kg, although considerable variance remained (comparing Figures 3g and 4b). Earlier, we identified a difference in the SHAP values of clearance for patients receiving treatment in center one. The SHAP analysis suggests that individuals without blood group O had SHAP values closer to zero compared to individuals with blood group O (Figure 4c). No such effect is seen for the other centers. For the SHAP values of blood group for clearance, we see a similar result. Here, individuals with lower body weight (65 kg) seem to have SHAP values closer to zero than those with higher body weight (Figure 4d).

A classical approach to obtain intuition on what functional forms to use would be to plot the empirical Bayes estimates of the PK parameters against each of the covariates. This visualization in shown in Figure S2. Here, we see that for highly correlated covariates (ie, weight), it is possible to derive some intuition on the functional form to use, but for most covariates their effect is difficult to discern. This is because we are unable to visualize the contribution of each covariate in isolation. Because unexplained residual variance is also present in the PK parameters, choosing a function to use can be more difficult due to large variation. This can mean that we have to iteratively select functions to implement covariates, reproduce the visualizations, and re‐evaluate, thus again resorting to a stepwise approach. With SHAP, we can decide on appropriate functions based on a single full model fit.

Although not shown, the functional forms of the covariates as described by the SHAP values of the two XGBoost models were very similar to those from the random forest. This suggested that the choice between a random forest and XGBoost had only minor effects on the subsequent SHAP analysis.

## DISCUSSION

In this study, we aimed to enrich ML‐based covariate selection methods using SHAP in order to infer the optimal function form to use when including covariates in PK models. We fit both a random forest and XGBoost model to predict empirical Bayes estimated PK parameters originating from a base NLME model. The random forest resulted in slightly more accurate PK parameter predictions compared to the XGBoost models. Next, influential covariates can, for example, be selected using importance scores.^5^ Finally, after performing a SHAP analysis, we are able to examine the relationship between each covariate and the PK parameters in greater detail. The SHAP analysis also allowed us to explore more complex interaction effects of covariates resulting from the sequential binning in tree‐based methods. Because SHAP values depict the absolute change in output value, the user can intuitively determine clinical relevance. These features display the benefit of SHAP values compared to using importance measures in isolation, where often only a single score of importance is obtained.

The SHAP analysis identified covariates that have previously been associated with the PK of FVIII concentrates. In addition, the suggested relationships of the covariates are similar to their implementation in previous PK models.^16^, ^20^, ^21^ First, we found that patient weight was the most important covariate to explain IIV for both clearance and central volume. The concept of allometric scaling is often applied to the relationship between weight and FVIII clearance. This is mirrored in the flattening of the SHAP values as weight increases (Figure 3a,g). As the central volume compartment represents the blood plasma, a relationship resembling a linear interaction with weight might be expected. An obvious exception exists for overweight individuals, which is represented by the SHAP values in the sharp decline in SHAP values seen for individuals with a BMI greater than 25 (Figure 3h). Measures of fat‐free mass have been suggested to better predict central volume, which could remove the need to model the effect of BMI.^22^

Next, we saw a negative interaction between age and clearance. This effect has been demonstrated before,^16^ and there might be multiple possible explanations for this effect. One such explanation is the finding that several blood coagulation factors, including VWF, increase with age.^23^, ^24^ It is well known that VWF binds to FVIII to protect it from degradation in the blood circulation. Similar to this effect, SHAP values for patients with blood group O depicted increased FVIII clearance, an effect likely linked to lower VWF:Ag levels seen in patients with blood group O.^25^ Looking at the interaction between blood group and weight (Figure 4d), we see that individuals below 65 kg (ie, usually younger individuals) with blood group non‐O have relatively higher clearance than heavier individuals. This might also be linked to the previously observed increase in VWF:Ag levels with age.^23^, ^24^ It is possible that weight was used by the random forest as a proxy for age. Higher VWF:Ag levels were also directly associated with a decrease in FVIII clearance by the model (Figure 3c). However, considering the large fraction of missing data (40.1%), a low number of patients at the extremes of VWF levels, and the fact that the measurements were outdated (ie, not measured during the surgical procedure) there remains uncertainty about the observed relationship between VWF:Ag and clearance. Interpreting the effects of covariates with large fractions of missing data should be handled with care.

The SHAP values indicate that individuals from center one had lower clearance compared to other centers. One possible explanation is the use of different assay reagents in this center. The results, however, also indicate that this effect is correlated with the patient blood group (Figure 4c). There could thus be some other factor influencing this effect. Because we worked with retrospective data, it is difficult to underpin the origin of this effect.

Finally, we notice an increase in clearance and central volume associated with patients who received BDD‐rFVIII concentrate. It is well known that use of BDD‐rFVIII leads to a underestimation of FVIII activity levels when using the one‐stage assay versus the chromogenic assay.^26^, ^27^ By changing the phospholipid source in the one‐stage assay, similar FVIII activity levels compared to the chromogenic assay are measured. This suggests that this effect is not due to increased clearance or distribution volume of BDD‐rFVIII.^27^ It is possible that this effect leaked into the PK parameter estimates (instead of being part of the estimated error) by the base NLME model. Most of its effect was on increasing the central volume estimate. This can be expected as it would lead to a decrease in predicted FVIII levels.

From the previous discussions, we see the possibility of identifying many subtle effects captured by the random forest model using SHAP. However, the method also has limitations. First, the quality of the empirical Bayes estimated PK parameters is an important factor affecting the accuracy of the ML model and quality of the SHAP analysis. In our case, this required fixing the residual error parameters and only including random effects on clearance and central volume. It might not be clear in advance what measures need to be taken to obtain reliable results. Inspecting the distribution of the resulting PK parameters and comparing these to prior results can be a way to decide on an effective strategy in obtain good quality PK parameter estimates.

Next, we used LOESS to obtain an average representation of the relationship between the covariates and PK parameters. Although this may be helpful for the identification of effects, it might also bias the user to find relationships that do not exist. The method might falsely represent the true effect when SHAP values have high variance or when data are sparse.

Another possible issue lies in the inclusion of covariates that displayed substantial fractions of missing values. For example, roughly 40% of VWF:Ag levels were missing. Although its relationship with clearance suggested by the SHAP values matches previous biological understanding, we might not want to include the covariate based on the current analysis alone. Previous studies have, however, included this covariate using a function matching the SHAP values.^20^, ^21^

A more general issue with the application of SHAP values in the context of PK models is that it results in an additive breakdown of the model. Often, covariate effects in PK models are instead implemented as a product of functions. This makes it difficult to compare the outcomes of SHAP analyses with classic methods of covariate analysis, such as forest plots obtained from an FFEM. In addition, by using products, we can prevent the PK parameters from becoming negative. However, because the relationships of the covariates suggested by the SHAP values match those used in previous PK studies, we assume that the functional forms might hold (up to a difference in parameters).^12^, ^14^, ^15^ Such an assumption will have to be validated.

Finally, although SHAP might be able to explain the covariate relationships in the ML model, this does not mean that the results are biologically interpretable. ML algorithms remain black box models, simply deconstructing the model in components does not guarantee that the results are humanly interpretable. For example, we found an effect of center one on clearance, which was correlated with patient blood group. With the current data we are unable to provide an explanation of this effect. Consequently, not every effect found by the SHAP analysis should necessarily be included in PK models.

In summary, we show that combining ML and SHAP allows for an in‐depth review of the relationships between covariates and PK parameters. We have mainly focused on using SHAP values for visualizing covariate relationships in ML models. SHAP values can also be used to perform covariate selection. Its benefit over importance scores will have to be evaluated. Covariate selection is a difficult issue, and our method is one of the first to allow one to infer the optimal function form to include covariates based on ML algorithms. The method can prove useful for covariate analysis and hypothesis generation.

## AUTHOR CONTRIBUTIONS

A.J., M.H., M.H.C., and R.A.A.M. wrote the manuscript. A.J., M.H., M.H.C., and R.A.A.M. designed the research. A.J. performed the research. A.J. analyzed the data.

## CONFLICT OF INTEREST

The authors declared no competing interests for this work.

## Supporting information


Figure S1

Figure S2

Appendix S1
