# Supplementary material for: Application of SHAP values for inferring the optimal functional form of covariates in pharmacokinetic modeling
Source: CPT Pharmacometrics Syst Pharmacol. 2022 Jun 24;11(8):1100–10. doi: 10.1002/psp4.12828 (PMC9381890; doi:10.1002/psp4.12828)

Figure S1. Covariate importance scores for the random forest model.
Permutation importance (white) and mean absolute SHAP value (black) scores for the clearance (A) and central volume (B) random forest models. The fraction of the total score is shown. Error bars for the permutation importance scores indicate the standard deviation of the importance scores from the ten models fit during cross validation.

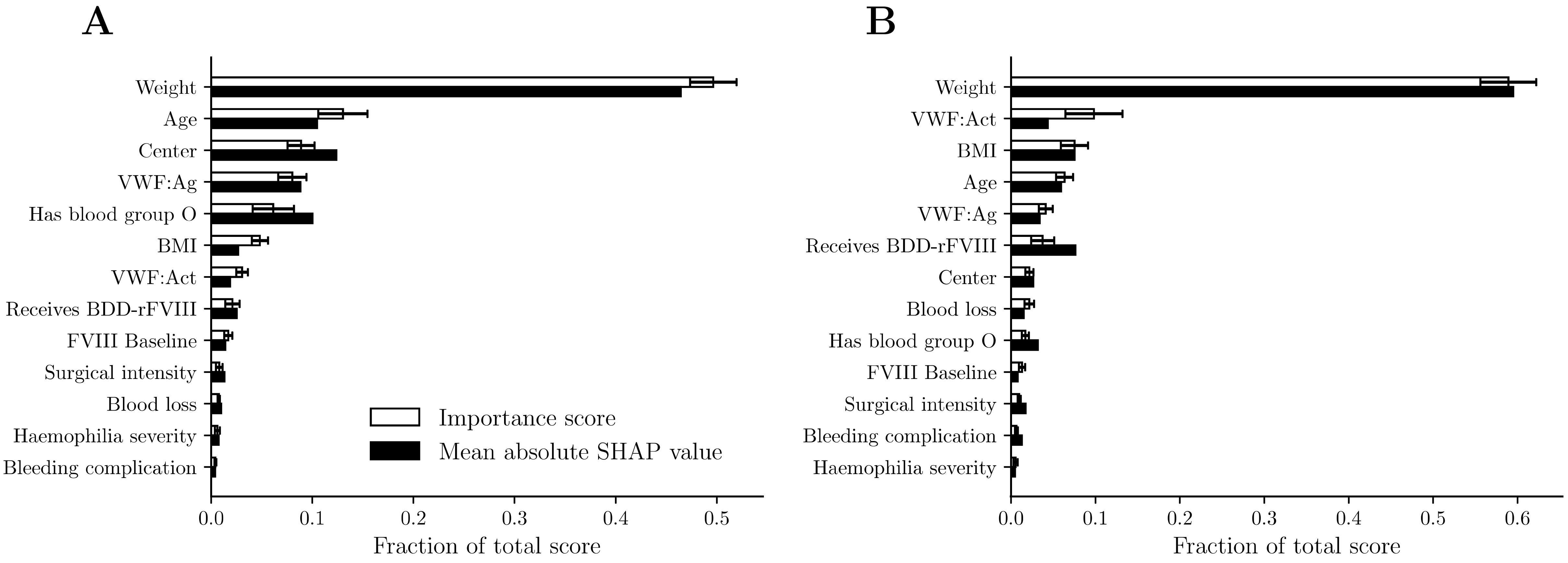

Supplement: Supplementary file 1 — Figure S1 [file PSP4-11-1100-s002.pdf]
