# Supplementary material for: Application of SHAP values for inferring the optimal functional form of covariates in pharmacokinetic modeling
Source: CPT Pharmacometrics Syst Pharmacol. 2022 Jun 24;11(8):1100–10. doi: 10.1002/psp4.12828 (PMC9381890; doi:10.1002/psp4.12828)
Supplement: Supplementary file 2 — Figure S2 [file PSP4-11-1100-s001.docx]

SUPPLEMENTARY MATERIALS

*S1 Default hyper-parameters for random forest and XGBoost models.*

Below we list the most important hyper-parameters (e.g. excluding those not influencing model performance such as logging etc.) of the random forest and XGBoost model. Hyper-parameter descriptions were directly obtained from the sci-kit learn documentation (https://scikit-learn.org/stable/modules/generated/sklearn.ensemble.RandomForestRegressor.html) and the XGBoost documentation (https://xgboost.readthedocs.io/en/stable/parameter.html).

Random forest model:

**n_estimators** = 100: The number of trees in the forest.

**max_depth** = None: The maximum depth of the tree. If None, then nodes are expanded until all leaves are pure or until all leaves contain less than min_samples_split samples.

**min_samples_split** = 2: The minimum number of samples required to split an internal node.

**min_samples_leaf** = 1: The minimum number of samples required to be at a leaf node. A split point at any depth will only be considered if it leaves at least *min_samples_leaf* training samples in each of the left and right branches.

**min_weight_fraction_leaf = 0.0: The minimum weighted fraction of the sum total of weights (of all the input samples) required to be at a leaf node. Samples have equal weight when sample_weight is not provided.**

**max_features** = “auto”: The number of features to consider when looking for the best split. If “auto”, then *max_features* = *n_features*.

**max_leaf_nodes** = None: Grow trees with max_leaf_nodes in best-first fashion. Best nodes are defined as relative reduction in impurity. If None then unlimited number of leaf nodes.

**min_impurity_decrease** = 0.0: A node will be split if this split induces a decrease of the impurity greater than or equal to this value.

**bootstrap** = true: Whether bootstrap samples are used when building trees. If False, the whole dataset is used to build each tree.

**max_samples** = None: If bootstrap is True, the number of samples to draw from X to train each base estimator. If None (default), then draw X.shape[0] samples.

**ccp_alpha** = 0.0: Complexity parameter used for Minimal Cost-Complexity Pruning. The subtree with the largest cost complexity that is smaller than *ccp_alpha* will be chosen. By default, no pruning is performed.

XGBoost model:

**booster** = “gbtree”: Which booster to use. Can be gbtree, gblinear or dart; gbtree and dart use tree based models while gblinear uses linear functions.

**eta** = 0.3: Step size shrinkage used in update to prevents overfitting. After each boosting step, we can directly get the weights of new features, and *eta* shrinks the feature weights to make the boosting process more conservative.

**gamma** = 0: Minimum loss reduction required to make a further partition on a leaf node of the tree. The larger gamma is, the more conservative the algorithm will be.

**max_depth** = 6: Maximum depth of a tree. Increasing this value will make the model more complex and more likely to overfit. 0 is only accepted in *lossguide* growing policy when *tree_method* is set as hist or *gpu_hist* and it indicates no limit on depth. Beware that XGBoost aggressively consumes memory when training a deep tree.

**min_child_weight** = 1: Minimum sum of instance weight (hessian) needed in a child. If the tree partition step results in a leaf node with the sum of instance weight less than *min_child_weight*, then the building process will give up further partitioning. In linear regression task, this simply corresponds to minimum number of instances needed to be in each node. The larger *min_child_weight* is, the more conservative the algorithm will be.

**max_delta_step** = 0: Maximum delta step we allow each leaf output to be. If the value is set to 0, it means there is no constraint. If it is set to a positive value, it can help making the update step more conservative. Usually this parameter is not needed, but it might help in logistic regression when class is extremely imbalanced. Set it to value of 1-10 might help control the update.

**subsample** = 1: Subsample ratio of the training instances. Setting it to 0.5 means that XGBoost would randomly sample half of the training data prior to growing trees. and this will prevent overfitting. Subsampling will occur once in every boosting iteration.

**colsample_bytree, colsample_bylevel, colsample_bynode** = 1: This is a family of parameters for subsampling of columns. All *colsample_by** parameters have a range of (0, 1], the default value of 1, and specify the fraction of columns to be subsampled.

*colsample_bytree* is the subsample ratio of columns when constructing each tree. Subsampling occurs once for every tree constructed.

*colsample_bylevel* is the subsample ratio of columns for each level. Subsampling occurs once for every new depth level reached in a tree. Columns are subsampled from the set of columns chosen for the current tree.

*colsample_bynode* is the subsample ratio of columns for each node (split). Subsampling occurs once every time a new split is evaluated. Columns are subsampled from the set of columns chosen for the current level.

**lambda** = 1: L2 regularization term on weights. Increasing this value will make model more conservative.

**alpha** = 0: L1 regularization term on weights. Increasing this value will make model more conservative.

**tree_method** = “auto”: The tree construction algorithm used in XGBoost. Choices: auto, exact, approx, hist, gpu_hist, this is a combination of commonly used updaters. For other updaters like refresh, set the parameter updater directly.

“auto”: Use heuristic to choose the fastest method. For small dataset, exact greedy (exact) will be used. For larger dataset, approximate algorithm (approx) will be chosen. It’s recommended to try hist and gpu_hist for higher performance with large dataset. (gpu_hist) has support for external memory.

**scale_pos_weight** = 1: Control the balance of positive and negative weights, useful for unbalanced classes. A typical value to consider: sum(negative instances) / sum(positive instances).

**num_parallel_tree** = 1: Number of parallel trees constructed during each iteration. This option is used to support boosted random forest.

**objective** = “reg:squarederror”: reg:squarederror: regression with squared loss.

**base_score** = 0.5: The initial prediction score of all instances, global bias
