# Supplementary material for: Application of SHAP values for inferring the optimal functional form of covariates in pharmacokinetic modeling
Source: CPT Pharmacometrics Syst Pharmacol. 2022 Jun 24;11(8):1100–10. doi: 10.1002/psp4.12828 (PMC9381890; doi:10.1002/psp4.12828)
Supplement: Supplementary file 3 — Appendix S1 [file PSP4-11-1100-s003.pdf]

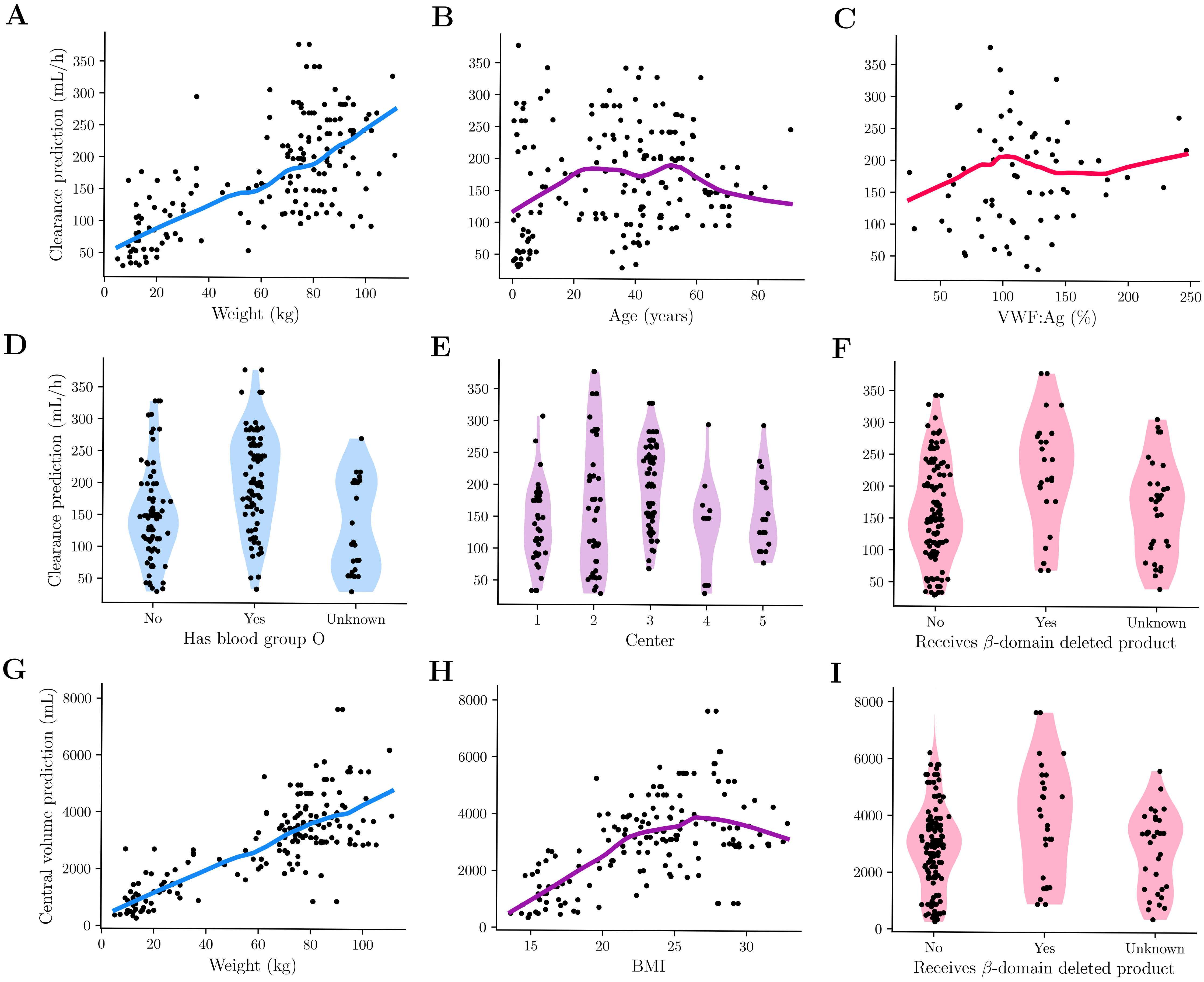

A
B
C
D
E
F
G
H
I
Clearance prediction (mL/h)
Weight (kg)
Age (years)
VWF:Ag (%)
Has blood group O
No
Yes
Unknown
Center
Receives β-domain deleted product
Central volume prediction (mL)
BMI

Figure S2. Correlation between covariates and PK parameter estimates.

Here the correlation between empirical Bayes estimates of the PK parameter and covariate values are shown. Points represents the PK parameter predictions, while lines indicate the LOESS fitted smooth representation of the relationship. For the categorical covariates the density is also shown by means of a violin plot. We again show the results for the most important covariates for clearance (A-F) and central volume (G-I).
